# Perspectives from the 2025 ISCBI/ISCI joint workshop on genetic stability, clonal monitoring, ethical data governance, and global inclusion in stem cell banking

**DOI:** 10.1186/s13287-025-04797-2

**Published:** 2026-01-03

**Authors:** Jung-Hyun Kim, Andreas Kurtz, Ivana Barbaric, Maneesha S. Inamdar, Martin F. Pera, Nissim Benvenisty, Nika Shakiba, Rosario Isasi, Tadaaki Hanatani, Glyn Stacey

**Affiliations:** 1 Department of Pharmacy, College of Pharmacy, Ajou University, Suwon, 16499 Gyeonggi-do South Korea; 2 Center for Regenerative Therapies, Berlin Institute of Health at Charite, Berlin, Germany; 3 The University of Sheffield, Sheffield, UK; 4 Jawaharlal Nehru Centre for Advanced Scientific Research (JNCASR), Bangalore, India; 5 Institute for Stem Cell Science and Regenerative Medicine (BRIC-inStem), Bangalore, India; 6 The Jackson Laboratory, Bar Harbor, ME USA; 7 The Azrieli Center for Stem Cells and Genetic Research, Department of Genetics, Silberman Institute of Life Sciences, The Hebrew University of Jerusalem, Jerusalem, Israel; 8 School of Biomedical Engineering, The University of British Columbia, Vancouver, BC Canada; 9 Premium Research Institute for Human Metaverse Medicine (PRIMe), The University of Osaka, Osaka, Japan; 10 Interdisciplinary Stem Cell Institute, John P. Hussman Institute for Human Genomics, University of Miami Leonard M. Miller School of Medicine, Miami, FL USA; 11 CiRA Foundation, Kyoto, 606-8397 Japan; 12 International Stem Cell Biobanking Initiative and Beijing Institute for Stem Cell Regenerative Medicine, Barley, Herts UK

## Abstract

Two international stem cell consortia, the International Stem Cell Initiative (ISCI) and the International Stem Cell Biobanking Initiative (ISCBI, www.iscbi.org) held a workshop on June 15th 2025 in Hong Kong on genetic variants in human pluripotent stem cell (hPSC) lines and accurate and standardized documentation of donor/hPSC genetic information including ethnicity. The occurrence and detection of genetic variants is a key issue for assuring reproducible stem cell research data and the safety of stem cell derived medicinal products. Presentations by leading experts addressed the nature of hPSC genetic variants, their detection and accurate recording of genetic data and ethnicity. The audience of stem cell researchers, cell banking directors and experts in ethic, policy and stem cell databases, from 13 countries across the globe, discussed progression of the ISCI consortium’s efforts in providing further data and thought leadership on the management of genetic variants, and the challenges for standardizing biobanking approaches for hPSC genetic data including ethnicity. This paper records the key elements of this discussion and the conclusions and consensus reached and ongoing work to provide guidance for hPSC biobanks.

## Introduction

**Dr. Glyn Stacey (Chair**,** ISCBI)**

Since its founding in 2007, the International Stem Cell Bioanking Initiative (ISCBI) (www.iscbi.org) [1–5] and the International Stem Cell Initiative (ISCI) [6–8], founded in 2003 have collaborated to serve as global platforms for harmonizing standards in human pluripotent stem cell (hPSC) research, banking, and clinical application. The 2025 joint meeting of these organizations. The meeting hosted at the Hong Kong Science and Technology Park (HKSTP), brought together experts from academia, industry, bioethics, and regulation.

The ISCI session chaired by Prof Ivana Barbaric (University of Sheffield, UK) addressed emerging trends and technologies for hPSC line genetic variants from the central themes of:

- Genetic stability and clinical safety in hPSC lines. How do we interpret the variants that we detect?
- Advanced clonal monitoring tools for manufacturing quality control.

The ISCBI session chaired by Profs Glyn Stacey and Rosario Isasi (University of Miami, USA) considered accurate assignment of stem cell genetic data from the perspectives of:

- Ethical and scientific frameworks for donor diversity and metadata.
- Data interoperability and global inclusion in stem cell registries.

This meeting report synthesizes the presentations and discussions outlining perspectives on stem cell banking.

##  Points to consider in genetic stability, ethical data governance and standardization of hPSC

### Prof. Nissim benvenisty (Hebrew University, Jerusalem, Israel)

#### Acquired cancer-related mutations in hPSC and implications for therapy

There are chromosomal and sub-chromosomal aberrations and copy number variations arising in hPSC in culture. In this meeting, Prof. Benvenisty focused on point mutations, especially cancer-related mutations. In a 2017 study published in *Nature* [9], the recurrent TP53 mutations in hPSC lines have been reported, demonstrating that these mutations conferred a strong selective advantage in vitro. All the mutations in *TP53* were at the DNA binding domain often found in cancer patients. In human embryonic stem cells (hESCs), almost 20% of them had a mutation in *TP53*. hPSC exhibited point mutations in multiple cancer-related genes, indicating culture adaptation in genomic integration outcomes.

In the presentation, Benvenisty detailed the results of a large-scale analysis (*Nature Biotechnology*, 2024) [10] of over 2,000 samples from 146 hPSC lines and their differentiated derivatives. Their study found that ~ 22% of samples harbored cancer-related mutations, most commonly in *TP53*, followed by other tumor suppressors and oncogenes. Among the mutations 60–70% are found in *TP53*. A follow up study demonstrated that unlike mutations in pluripotent stem cells, in mesenchymal and neural stem cells, *TP53* is not the most prevalent mutated gene. This reflects what is seen in tumors: *TP53* is most frequent in teratoma and teratocarcinoma, but not in undifferentiated sarcomas or neural tumors. Mutations in cancer genes can take over, especially with bottlenecks or clonality. Loss of heterozygosity was observed in tumor suppressor genes, indicating clonal dominance.

Functionally, *TP53* mutations impaired differentiation kinetics, especially in neural induction, and allowed persistence of markers associated with pluripotency. These data align with the U.S. FDA’s 2024 draft guidance, which recommends ≥ 50× whole-genome sequencing not only for cytogenetic screening but also for reporting all cancer-associated mutations, with particular concern regarding *TP53* [11].

- *Key perspective* High-coverage genomic screening, combined with “stress-testing” of master cell banks through extended culture, are essential to detect low-frequency mutant subclones prior to clinical application.
- *Key perspective* High-coverage genomic screening, combined with “stress-testing” of master cell banks through extended culture, is essential to detect low-frequency mutant subclones prior to clinical application.

### Prof. Nika Shakiba (University of British Columbia, Canada)

#### DNA barcoding for high-resolution tracking of clonal dynamics

Prof. Shakiba introduced DNA barcoding as a powerful tool for understanding heterogeneity and the emergence of variants in stem cell manufacturing systems, from both a basic perspective and from a manufacturing and quality control perspective [12]. DNA barcoding involves integration of a short DNA sequence into the genome of a cell, either randomly or into safe harbor sites. Once integrated, barcoded cells propagate and all progeny carry the same barcode. This allows tracking of clonal dynamics through expansion or differentiation. By using DNA barcodes as proxies for growth, one can identify clones outgrowing the population and assess whether they were abnormal from the start or acquired abnormalities. DNA barcodes in human embryonic stem cells can be used for naive induction as a proof of concept. Prof. Shakiba highlighted recent work that showed that subsets of clones robustly underwent naive transition in parallel cultures, identifying elite clones that were not dominant in primed populations but special in naive transition. This highlights the ability of DNA barcoding to reveal hidden heterogeneity.

Adapted from tumor evolution studies, this method offers several benefits for regenerative medicine:

- Identifying “elite” clones with disproportionate survival or proliferation.
- Linking barcode identity to genomic and phenotypic data by preserving archived cell stocks.
- Modeling the effects of manufacturing perturbations on clonal competition.

Recent developments allow retrospective isolation of clones based on their barcodes. By targeting the barcode region with CRISPR, clones can be isolated from frozen stocks to ask whether hidden genetic or epigenetic abnormalities existed. This can also be extended with GFP reporters or even kill switches to selectively remove problematic clones.

DNA barcoding could be used internationally to deploy high-complexity barcoded hPSC libraries across labs. Each lab could passage, differentiate, and then sequence barcodes to track selection pressures. Loss of barcodes or overgrowth of certain clones would reveal selection dynamics. This can benchmark bioprocesses and test whether selective practices accelerate variant emergence.

Another proposal is to use DNA barcoding with libraries of genetic variants, either naturally emerged or engineered. By expanding and differentiating these barcoded variants, labs can identify which variants survive under which conditions and, understand the underlying mechanisms that confers their survival, with implications for cell therapies.

A further idea is to include DNA barcoding directly in clinical lines, using them as analytical tools to track selection and dominant clones during manufacturing as part of in process quality control.

### Prof. Rosario Isasi, J.D., M.P.H. (Dr. John T. Macdonald foundation department of human Genetics, university of Miami, USA)

#### Beyond race and ethnicity: scientific and ethical imperatives for donor metadata

Rosario Isasi, legal scholar and bioethicist, examined the scientific and ethical challenges of how population descriptors are currently applied in donor metadata. She underscored the inadequacy of race and ethnicity categories—socially constructed rather than biologically precise—as stand-ins for genetic diversity. Building on guidance from the *National Academies of Sciences*,* Engineering*,* and Medicine* (NASEM*)* [13] along with international policy frameworks, she advocated for approaches outlined below.

Guidance for researchers emphasizes that descriptors for populations must be tailored to the context of the study. There is no universal standard; strict standardization is not recommended, but rather harmonization. Transparency and rigor in documenting and justifying the use of labels are important, balancing self-identified race and ethnicity versus genomic variations. The relevance of population descriptors depends on the study: they may not be necessary for trait prediction, but are pertinent in health disparities where genetic, epigenetic, and environmental factors interact.

Currently, racial, ethnic, or continental ancestry are often misused as proxies of genetic variation, but they are poor proxies without scientific rigor. Labels are sociopolitical constructs that conflate social identity with biology, perpetuate biological essentialism, and have ethical implications. Guidelines recommend abandoning typology thinking and adopting descriptors with more genetic and contextual precision, prioritizing genomic ancestry at the individual level when justified, and reporting ancestry only when relevant. Quantitative measures of genetic similarity should be used rather than social labels.

Phenotypes must be contextualized, integrating environmental and social experience. Donors carry epigenetic signatures shaped by ancestry and environmental exposures. Wrong typology obscures determinants such as stress, nutrition, and socioeconomic diversity. Ancestry should not be treated as a full biological explanation. Banking and registries must consider whether population labels are self-reported, who reported them, and why they are included, leaving nuance for donor-centered descriptors. Communities must be engaged to inform labeling, consent, and governance.

Ethical considerations include collective risk. Consent is a process, not a document, and standard consent often lacks coverage of ancestry and data sharing. Dynamic consent models are needed. Risks of labeling include stigmatization and discrimination, discouraging participation. Misattributing genetic attributions to race and ethnicity creates racism, as seen in flawed studies linking population labels with height, dementia, or education. Often the causes are environmental and socioeconomic, not genetic.

The NASEM recommendations highlight ten principles, emphasizing rigor in population descriptors for donors and stem cell research. Isasi proposed that pathways forward include training tools, evidence-based guidelines, ontologies, and interdisciplinary collaboration to monitor uptake and impact across research programs.

### Prof. Andreas Kurtz (Berlin Institute of health at Charité)

### Data standards, interoperability, and the role of registries

Dr. Kurtz reviewed the metadata collection of global registry for human pluripotent stem cell lines (hPSCreg) [6, 14] with a focus on genetic diversity and ethnicity, and highlighted ongoing challenges to obtaining and making this data available in a findable, accessible, interoperable and reusable (FAIR) manner.

Fewer than 10% of hPSC - lines registered in hPSCreg include ancestry or ethnicity data, → and those that do are mostly donor self-assigned and ambiguous. Dynamic consent for continuous feedback from donors could be used for verification of genetic diversity and ancestry, however this is not implemented in stem cell banks and related data resources. A registry of cell lines could enable dynamic consent by implementing a trusted communication and verification platform without compromising donor identity.

Genetic metadata (Short tandem repeat - STR, Human Leukeucyte Antigen - HLA profiles) are suitable to assess cell line authenticity and most hPSCreg – lines are annotated. However, they are only partially suited as classifiers of genetic diversity. Genomic/transcriptomic sequencing data suitable as classifiers of genetic diversity are available for hPSC – lines registered in hPSCreg via access-controlled links to this personal data. However, linking hPSC – lines to genetic data deposited in federated data resources such as the European Bioinformatics Institute (EBI), or publications is often hampered by lack of findability via a matching identifier.

The inconsistent use of identifiers leads to lack of findability of metadata including those related to genetic diversity, duplication of data records, inconsistencies across databases and ambiguous digital cell phenotypes. The hPSCreg attempt to match and link diverse identifiers for metadata of diverse platforms is a possible approach, but bears risks for mismatching and requires persistence of all platforms.

It was advocated for the strict application of FAIR principles to make data findable and interoperable. Persistent identifiers linked to structured (e.g. ontology-based) metadata, enabling federated, privacy-preserving data sharing allows accessing genetic data from diverse resources and provide an applicable framework for assessing genetic diversity of hPSC - lines. Dynamic consent models could allow for ongoing donor governance while accommodating diverse legal environments for data use and exchange. Drawing on Global Alliance for Genomics and Health (GA4GH) standards. It was stressed that technical interoperability must be matched by semantic harmonization for true global data integration.

### Regional perspectives: India, Japan, and Korea

*India – Prof. Maneesha S Inamdar (InStem)* The Institute for Stem Cell Science and Regenerative Medicine (BRIC-inStem) in Bangalore is building local capacity by connecting academia and industry, focusing on Indian genetic diversity in pluripotent stem cell lines with pipelines for quality control and banking. The inStem Pluripotent Stem cell bank (inStem PluS) has human ESC lines [15], iPSC lines from controls and patients with mental and neurological disorders, and HLA-matched “GMP-grade” lines. HLA haplotype lines from 235 donors include 15 GMP iPSC lines now in seed stocks. These resources, over 100 lines research-grade and GMP-grade, can support disease modeling, drug screening, and early development models, and will be expanded for diversity and made available.

*Japan – Dr. Tadaaki Hanatani (CiRA Foundation)* In a relatively homogeneous population, such as Japan, ethnicity is seldom recorded; and at the CiRA foundation the focus is on GMP-compatible genomic testing aligned with Japanese regulation, U.S. FDA’s 2024 draft guidance and ISSCR guidelines.

*Korea – Prof. Jung-Hyun Kim (Ajou University)* Former director of the Korean National Stem Cell Bank, Kim described large-scale NGS datasets from banked lines, including the discovery of an Asian-specific CNV with functional implications, highlighting the need for the region-specific genetic ethnicity on recurrent CNV in hPSC to be characterized.

## Cross-cutting themes and provisional recommendations

The delegates concurred that there were a number of key themes and provisional recommendations that had emerged in the ISCI and ISCBI open discussion sessions.

### Emerging trends and technologies

#### Strengthen genetic quality control

Routine, high-coverage whole genome sequence (WGS) with standardized interpretation pipelines.

Long-term culture expansion tests to expose rare mutant clones.

#### Integrate clonal monitoring

Combine DNA barcoding with genomic and phenotypic assays for predictive manufacturing control.

### Accurate genetic assignment of hPSC lines

#### Ethically robust metadata

Replace race/ethnicity labels with genomic diversity metrics contextualized by environmental data.

Use donor-centered dynamic consent frameworks.

#### Enhance registry interoperability

Adopt persistent identifiers and harmonized ontologies across databases.

Enable federated data sharing while protecting privacy.

#### Expand global inclusion

Increase representation from under-sampled populations.

Integrate regional banks into global collaborative networks.

## Development of an ISCBI consensus on accurate Documentation of genetic and ethnic data for hPSC biobanks

During the workshop various standards for different aspects of stem cell genetic data were discussed. It appeared that there were examples of well- established standards that could be used for hPSC research and some have been actively adopted successfully. However, there were also aspects of stem cell genetic data where there was a clear need for a consensus on what standards should be recommended for use in hPSC biobanks and other aspects where a “standard” was not needed or could be unhelpful due to rapid developing scientific knowledge. One area identified where a consensus was needed to help improve and standardize biobank nomenclature was the ethnicity of cell lines. Currently this was largely self- assigned by donor statements but workshop participants concluded that some form of genealogical reporting mechanism was needed. It was recognized that this could be challenging to implement as such criteria may clash with donor perceptions and beliefs.

At the ISCBI–ISCI meeting, we discussed the genetic stability and clinical safety of hPSC lines from multiple perspectives. We still do not fully understand the correlations between genotype and phenotype, nor can we clearly distinguish which variants pose risks and which do not. Importantly, it is not yet possible to interpret the significance of all SNPs. However, large-scale global studies conducted previously have identified recurrent variations in hPSCs.

At this stage, both the MFDS (Korea’s Ministry of Food and Drug Safety) and Japan’s regulatory body have recommended evaluating mutations in oncogenic genes, including TP53. There is a clear need to develop more standardized methods and readout criteria for detecting genetic variants.

We recognize that stem cells are defined by their pluripotency and undifferentiated state, and that genetic alterations during culture can affect their phenotype and behavior. Therefore, the consensus at the meeting was that stem cell stocks and cultures should be routinely monitored for culture-acquired genetic changes. Evaluations of master and working cell banks are recommended to assess their genetic status throughout the experimental process—before initiation, during culture, and after major culture bottlenecks. However, the significance of such genetic variants in terms of patient safety is yet to be established and requires informative in vitro assays to assist in product safety testing.

It was agreed by delegates that it would be helpful to launch a joint ISCBI and ISCI activity to investigate these issues discussed in more detail and establish consensus on good practice in recording and documenting genetic information. This would be delivered by forming an expert working group selected from the ISCI and ISCBI communities and other invited experts. This group was charged with developing the current provisional conclusions from the workshop to consider what standards should be adopted by hPSC biobanks and resolve a consensus document for hPSC biobanking and this would be submitted for publication in a leading stem cell journal.

## Conclusion

### Glyn Stacey (Chair, ISCBI) and Ivana barbaric (Chair ISCI)

The 2025 ISCBI/ISCI meeting reinforced that new technologies—such as high-resolution clonal monitoring and standardized genomic stability pipelines—will be critical to ensuring the safety of hPSC-based therapies. Ethical frameworks that move beyond race and ethnicity toward precise, context-rich metadata will enhance both scientific accuracy and social responsibility. Finally, harmonized identifiers and interoperable registries will enable the global collaboration necessary to develop diverse, safe, and effective regenerative medicine products. It was hoped that the consensus proposed at this workshop would form a valuable assistance to the hPSC biobanking community and stem cell researchers.

## Data Availability

No datasets were generated or analysed during the current study.
